# Changes in Plasma Metabolomic Profile Following Bariatric Surgery, Lifestyle Intervention or Diet Restriction—Insights from Human and Rat Studies

**DOI:** 10.3390/ijms24032354

**Published:** 2023-01-25

**Authors:** Ilja Balonov, Max Kurlbaum, Ann-Cathrin Koschker, Christine Stier, Martin Fassnacht, Ulrich Dischinger

**Affiliations:** 1 Department of General, Visceral, and Transplant Surgery, Ludwig-Maximilians-University Munich, 81377 Munich, Germany; 2 Department of Internal Medicine I, Division of Endocrinology and Diabetes, University Hospital, University of Wuerzburg, 97080 Wuerzburg, Germany; 3 Central Laboratory, Core Unit Clinical Mass Spectrometry, University Hospital, University of Wuerzburg, 97080 Wuerzburg, Germany; 4 Department of General and Visceral Surgery, Sana-Krankenhaus Huerth GmbH, 50354 Huerth, Germany

**Keywords:** metabolomics, phosphatidylcholines, sphingolipids, branched-chain amino acids, obesity, Roux-en-Y Gastric Bypass, rodent model, insulin resistance

## Abstract

Although bariatric surgery is known to change the metabolome, it is unclear if this is specific for the intervention or a consequence of the induced bodyweight loss. As the weight loss after Roux-en-Y Gastric Bypass (RYGB) can hardly be mimicked with an evenly effective diet in *humans*, translational research efforts might be helpful. A group of 188 plasma metabolites of 46 patients from the randomized controlled Würzburg Adipositas Study (WAS) and from RYGB-treated *rats* (*n* = 6) as well as body-weight-matched controls (*n* = 7) were measured using liquid chromatography tandem mass spectrometry. WAS participants were randomized into intensive lifestyle modification (LS, *n* = 24) or RYGB (OP, *n* = 22). In patients in the WAS cohort, only bariatric surgery achieved a sustained weight loss (BMI −34.3% (OP) vs. −1.2% (LS), *p* ≤ 0.01). An explicit shift in the metabolomic profile was found in 57 metabolites in the *human* cohort and in 62 metabolites in the rodent model. Significantly higher levels of sphingolipids and lecithins were detected in both surgical groups but not in the conservatively treated *human* and animal groups. RYGB leads to a characteristic metabolomic profile, which differs distinctly from that following non-surgical intervention. Analysis of the *human* and *rat* data revealed that RYGB induces specific changes in the metabolome independent of weight loss.

## 1. Introduction

The prevalence of obesity has nearly tripled in the last 40 years and continues to increase in many parts of the world [1]. Recent studies report at least 30% of men and 35% of women to be obese in the US [2]. As a result, obesity and related metabolic disorders, particularly type 2 diabetes, are responsible for more deaths than undernourishment in most countries of the world [1]. Insulin resistance (IR), cardiovascular diseases and musculoskeletal disorders [3,4] as well as several types of cancer are well-known comorbidities of obesity [3,5,6]. Bariatric surgery, such as Roux-en-Y Gastric Bypass (RYGB), has proven not only to achieve a sustained weight loss but also a remission of diabetes and an improvement in other comorbidities [7,8].

Obesity correlates with an alteration in the small molecules in the fluids and tissues of the body [9,10]. The composition of these circulating small molecules or metabolites, the so-called metabolome, changes with obesity itself and again with an effective treatment [11]. It is well known that metabolomic profiles consisting of acylcarnitines (C), phosphatidylcholines (PC), lysophosphatidylcholines (lysoPC), sphingolipids (SM), amino acids, sugars and nucleotides correlate with lifestyle factors in general [9,12]. Blood metabolomic profiles of obesity and IR were characterized in multiple observation studies [13,14]. Notably a profound shift in SMs, PCs and amino acids after bariatric surgery has been repeatedly described [4,15]. Furthermore, associations between the branched-chain amino acids (BCAA), isoleucine, leucine and valine and the body mass index (BMI) were consistently reported [16]. BCAA have been described previously as potential biomarkers for IR [17,18]. Although the pathophysiological background of most of these metabolites has been shown before, the knowledge about causality and translational relevance is still poorly understood [11,19,20]. One reliable method to measure targeted metabolomics is liquid chromatography coupled with tandem mass spectrometry (LC-MS/MS) [21]. This high-throughput analysis allows the quantification of large numbers of low-molecular-weight metabolites in fluids, cells or tissue [5,11].

To identify metabolomic changes induced by RYGB, serum samples of morbidly obese patients treated with bariatric surgery (OP) or psychotherapy-enhanced lifestyle intervention (LS) from the randomized controlled Würzburg Adipositas Study (WAS) were analyzed as well as samples of *rats* treated with RYGB and body-weight-matched controls under a restrictive diet [22].

## 2. Results

### 2.1. Participants from the WAS Trial

Characteristics of the patient cohort at the start of the study have been described in detail previously [22]. At baseline, the patients had a mean BMI of 48 kg/m^2^. Further clinical characteristics are presented in Table 1. Significant differences could be detected in thirteen metabolites between both groups. No biochemical pathway could be identified with these thirteen metabolites via principal component analysis (see Supplementary Figure S1).

At 1-year follow-up patients of the RYGB-surgery group achieved a significant weight loss of −34.3% (BMI 49.1 [46–51] kg/m^2^ vs. 32 [30–33] kg/m^2^, *p* < 0.01) as well as a significant improvement in IR measured by the Homeostatic Model Assessment for Insulin Resistance (HOMA-IR) (5.1 [4.6–6.1] vs. 1.4 [1–1.9], *p* < 0.01). In contrast, a non-significant weight loss of −1.2% (BMI 48.2 [45–50] vs. 47.5 [45–49] kg/m^2^, *p* = 0.07) was detected in the LS group one year after randomization. Accordingly, there was no significant change in the HOMA-IR (7.5 [6.7–7.8] vs. 7.5 [3.3–7.8], *p* = 0.1). Apart from the gender distribution, no significant differences in clinical parameters could be detected at baseline (see Table 1).

A total of 188 metabolites were measured in 46 serum samples at randomization and one year after randomization. Forty-nine metabolites were not considered due to exclusion criteria. Thus, 139 metabolites (9 Cs, 21 amino acids, 9 biogenic amines, 13 lysoPCs, 72 PCs, a hexose and 14 SMs) were valid and retained for the main analysis and the derivation of metabolomic profiles. Fifty-seven metabolites were found to be significantly different between groups (1 acylcarnitine, 13 amino acids, 1 biogenic amine, 5 lysoPCs, 31 PCs, 5 SMs and a hexose) as shown in Table 2.

**Figure 1:**
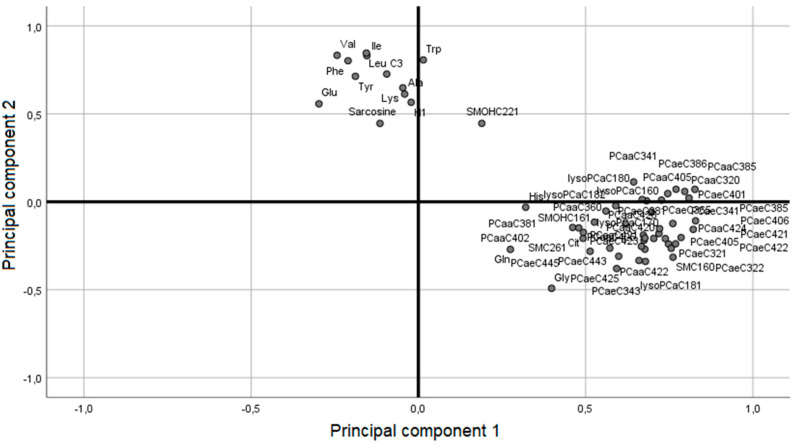
Unsupervised principal component analysis of 57 significantly different metabolites between lifestyle intervention and surgery group in the WAS cohort at 1-year follow-up. The first component explains 32% of the variation, and the second component 17%. C, acylcarnitine; lysoPC, lysophosphatidylcholine; PC aa, phosphatidylcholine with ester bonding; PC ae phosphatidylcholine with ether bonding; SM, sphingolipid.

The performed principal component analysis (PCA) resulted in twelve discriminating components with an eigenvalue > 1. The first two components explained 49% of the variance. Hence, further investigation was performed based on a two-component model. The first principal component (PC1) was characterized predominantly by phosphatidylcholines and sphingolipids, whereas the second principal component (PC2) was characterized by BCAA, aromatic acids and acylcarnitines (see Figure 1).

**Figure 2:**
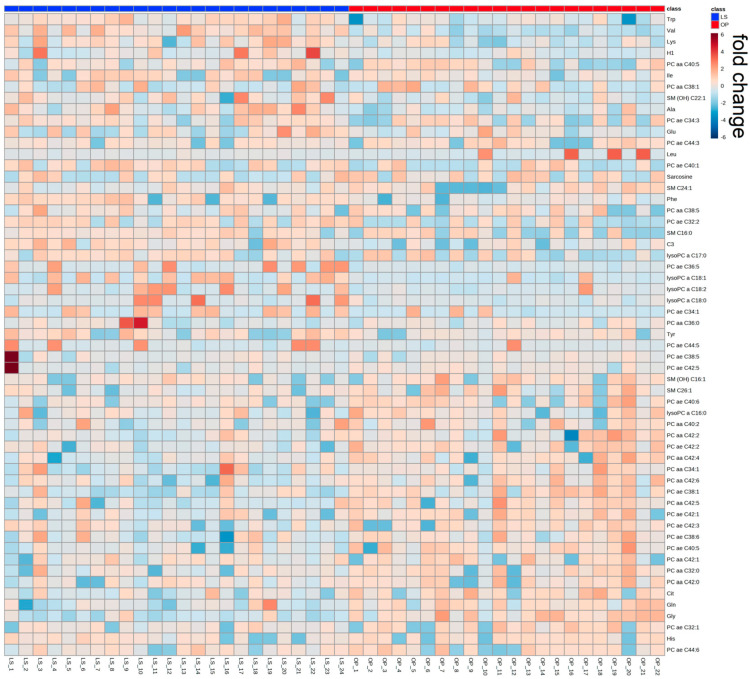
Heat map showing the scaled relative abundance of metabolites, which were significantly different between LS and OP group at one-year follow-up, visualized by hierarchical clustering. Abundance values represent row-wise z-scores of read counts. Each bar in the horizontal columns (which represent different patients) represents the expression intensity. Blue indicates a decreased level, red indicates an increased level. C, acylcarnitine; lysoPC, lysophosphatidylcholine; PC aa, phosphatidylcholine with ester bonding; PC ae phosphatidylcholine with ether bonding; SM, sphingolipid.

We performed hierarchical clustering of the 57 metabolites with significant differences between the LS and OP group (see Figure 2) at one-year follow-up. A cluster of phosphatidylcholines, especially PC aa C42:Ys, as well as in SM (OH) C16:1, SM C26:1, lysoPC a C16:0, glutamine, glycine, citrulline and histidine were identified to be enriched only in patients from the OP group but not in patients from the LS group.

### 2.2. Rodent Model

Characteristics of the rodent model have been described in detail previously [23]. Rats of the bariatric surgery group (RYGB_rat) experienced a significant weight loss of −7.3% (486.8 ± 9.3 g vs. 451.3 ± 18.5 g). In the body-weight-matched group (BWM_rat), a weight loss of 1.1% (481.9 ± 12.7 g vs. 476.4 ± 28.8 g) could be detected. At the time of the blood sampling, the body weights of RYGB_rat and BWM_rat were not significantly different (*p* = 0.16). A total of 188 metabolites were measured. Fifty-seven metabolites were excluded due to the below-mentioned criteria. A total of 131 metabolites (6 Cs, 19 amino acids, 10 biogenic amines, 13 lysoPCs, 68 PCs, a hexose and 14 SMs) were valid and retained for the main analysis and the derivation of metabolomic profiles. Sixty-two metabolites were found to be significantly different as shown in Table 3.

**Figure 3:**
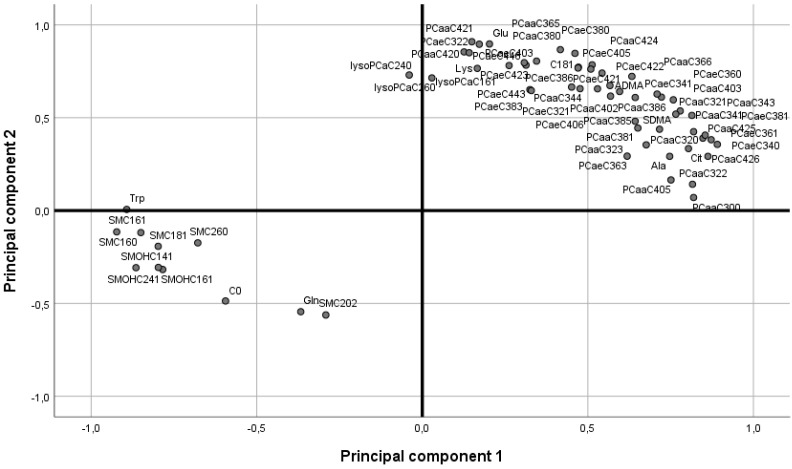
Unsupervised principal component analysis of significantly different metabolites (n = 62) between BWM and RYGB rats. The first component explains 52% of the variation, and the second component 22%. C, acylcarnitine; lysoPC, lysophosphatidylcholine; PC aa, phosphatidylcholine with ester bonding; PC ae phosphatidylcholine with ether bonding; SM, sphingolipid.

Further analysis of these metabolites (2 acylcarnitines, 6 amino acids, 2 biogenic amine, 3 lysoPCs, 41 PCs and 8 sphingolipids) with PCA showed that the first two components explained 74% of the variance. PC1 was mainly characterized by phosphatidylcholines. PC2 was not characterized by a specific subgroup of metabolites (see Figure 3) four weeks after the start of the interventions.

**Figure 4:**
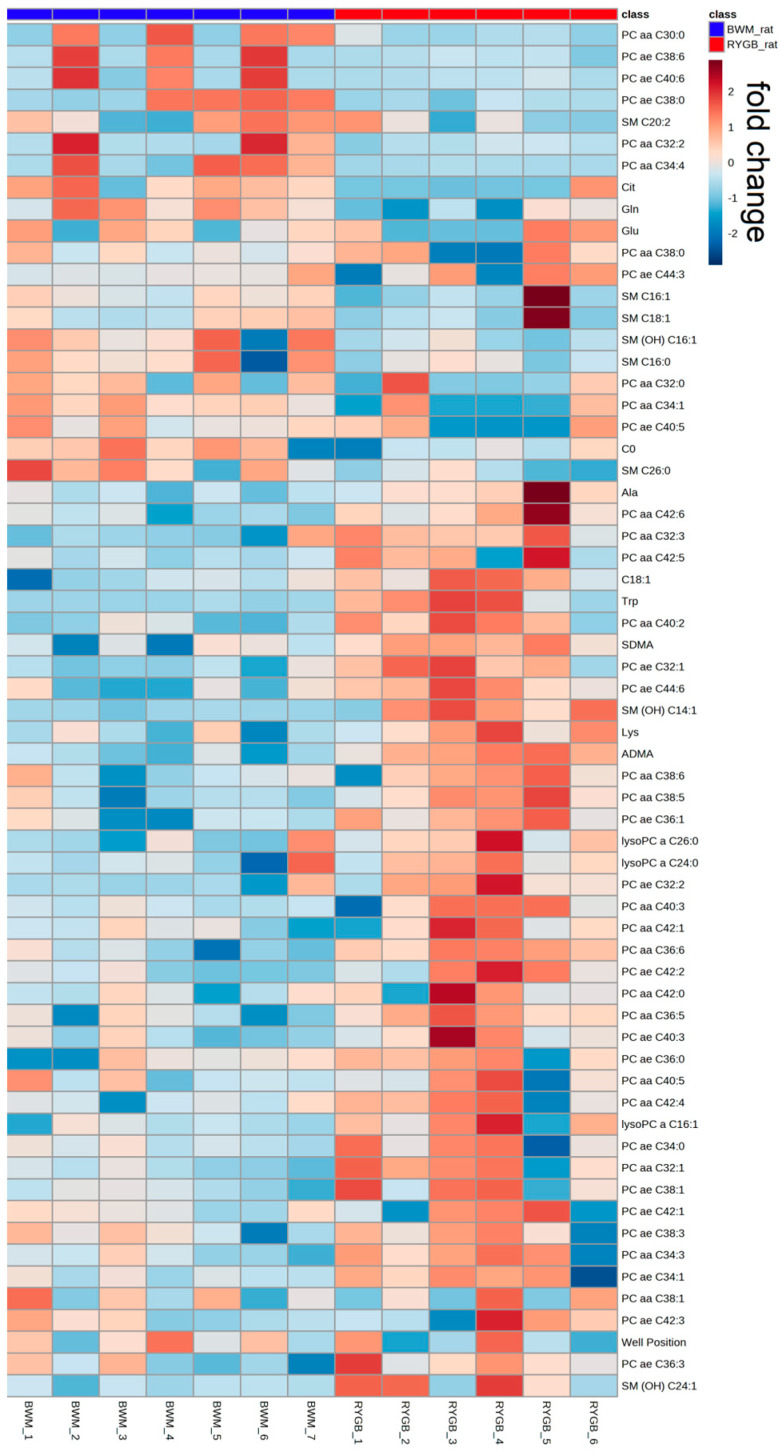
Heat map with the scaled relative abundance of 62 significantly different metabolites in BWM and RYGB rats four weeks postoperative visualized by metabolite hierarchical clustering. Abundance values represent row-wise z-scores of read counts. Each bar in the horizontal columns (which represent different rats) represents the expression intensity. Blue indicates a decreased level, red indicates an increased level. C, acylcarnitine; lysoPC, lysophosphatidylcholine; PC aa, phosphatidylcholine with ester bonding; PC ae phosphatidylcholine with ether bonding; SM, sphingolipid.

We performed hierarchical clustering of the 62 metabolites with significant differences between the RYGB_rat and the BWM_rat group (see Figure 4). A cluster of phosphatidylcholines with ester and ether bonding, especially PC aa C42:Ys, as well as in SM (OH) C14:1, SM (OH) C24:1, lysoPC a C16:1, lysine, tryptophane, alanine and in the biogenic amines ADMA and SDMA were identified as being enriched only in the RYGB_rat group but not in the weight-identical BWM_rat group.

### 2.3. Analysis of Overlapping Metabolomic Profiles in the Human OP and the Rat RYGB Group

**Figure 5:**
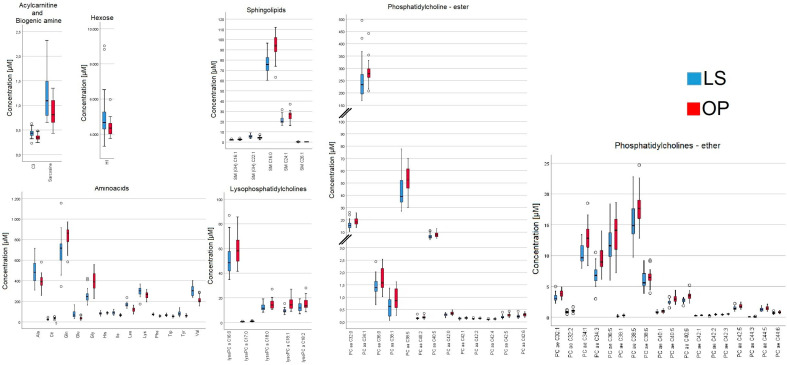
Metabolomic profile of 57 significantly different metabolites of acylcarnitines; amino acids; lysophosphatidylcholines; sphingolipids; acylcarnitines; biogenic amines; phosphatidylcholines with ester bonding; phosphatidylcholines with ether bonding; hexose, between lifestyle (LS) and RYGB (OP) group at one-year follow-up.

**Figure 6:**
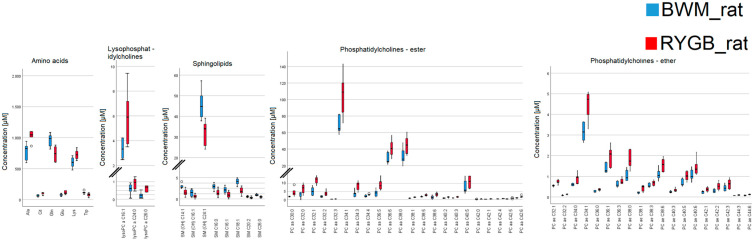
Metabolomic profile of 62 significantly different parameters of amino acids; lysophosphatidylcholines; sphingolipids; acylcarnitines; phosphatidylcholines with ester bonding; phosphatidylcholines with ether bonding, between rats with RYGB and rats with diet restriction (BWM) four weeks after start of the interventions.

Thirty-one metabolites with significant differences in both OP vs. LS and RYGB_rat vs. BWM_rat could be identified (see Table 4). Additionally, we performed an indirect comparison between the *human* data and the rodent model. In accordance with previously published data, the transferability of data from a morbidly obese patient to a rat with diet-induced obesity has to be performed with caution and only in cohesion with methodical comparability (e.g., comparable surgery) [24]. In indirect comparison to the conservatively treated groups LS and BWM_rat, the surgical groups OP and the RYGB_rat were characterized by metabolomic profiles of increased long-chain phosphatidylcholines and citrulline as well as decreased tryptophan (see Figure 5 and Figure 6). The data are presented as means with standard deviation, respectively.

In terms of individual metabolites, six amino acids with a significant difference between RYGB_rat and BWM_rat were also found to be significantly different in the *human* cohort: Alanine (485.6 µM in LS vs. 392.6 µM in OP; 829 µM in BWM_rat vs. 1044.5 µM in RYGB_rat), citrulline (25.5 µM in LS vs. 35.8 µM in OP; 73.3 µM in BWM_rat vs. 123.5 µM in RYGB_rat), glutamine (702 µM in LS vs. 834 µM in OP; 946 µM in BWM_rat vs. 735.5 µM in RYGB_rat), glutamate (73.7 µM in LS vs. 38.5 µM in OP; 67 µM in BWM_rat vs. 99.2 µM in RYGB_rat), lysine (296.9 µM in LS vs. 260.5 µM in OP; 602 µM in BWM_rat vs. 722.5 µM in RYGB_rat) and tryptophan (65.5 µM in LS vs. 54.5 µM, in OP; 113 µM in BWM_rat vs. 82.3 µM in RYGB_rat).

Analyzing the phosphatidylcholines with ester bonding, eleven PCs could be identified to be significantly different in both comparisons: PC aa C32:0 (16.0 µM in LS vs. 18.5 µM in OP; 5.7 µM in BWM_rat vs. 11.4 µM in RYGB_rat), PC aa C34:1 (257 µM in LS vs. 283 µM in OP; 66 µM in BWM_rat vs. 106 µM in RYGB_rat), PC aa C38:1 (0.62 µM in LS vs. 0.97 µM in OP; 0.43 µM in BWM_rat vs. 1.08 µM in RYGB_rat), PC aa C38:5 (44.1 µM in LS vs. 52.1 µM in OP; 24.3 µM in BWM_rat vs. 40.5 µM in RYGB_rat), PC aa C40:2 (0.16 µM in LS vs. 0.19 µM in OP; 0.26 µM in BWM_rat vs. 0.51 µM in RYGB_rat) and PC aa C40:5 (6.5 µM in LS vs. 8.0 µM in OP; 5.7 µM in BWM_rat vs. 9.2 µM in RYGB_rat).

Five of these PCs are characterized by forty-two carbon atoms (PC aa C42:Y): PC aa C42:0 (0.29 µM in LS vs. 0.35 µM in OP; 0.06 µM in BWM_rat vs. 0.10 µM in RYGB_rat), PC aa C42:1 (0.13 µM in LS vs. 0.16 µM in OP; 0.08 µM in BWM_rat vs. 0.11 µM in RYGB_rat), PC aa C42:4 (0.09 µM in LS vs. 0.12 µM in OP; 0.13 µM in BWM_rat vs. 0.20 µM in RYGB_rat), PC aa C42:5 (0.21 µM in LS vs. 0.28 µM in OP; 0.13 µM in BWM_rat vs. 0.28 µM in RYGB_rat) and PC aa C42:6 (0.24 µM in LS vs. 0.29 µM in OP; 0.31 µM in BWM_rat vs. 0.49 µM in RYGB_rat).

Twelve phosphatidylcholines with ether bonding were found to be significantly different in the *human* cohort, ten of these phosphatidylcholines were significantly different in RYGB_rat vs. BWM_rat: PC ae C32:1 (3.18 µM in LS vs. 3.85 µM in OP; 0.54 µM in BWM_rat vs. 0.72 µM in RYGB_rat), PC ae C32:2 (0.85 µM in LS vs. 1.04 µM in OP; 0.09 µM in BWM_rat vs. 0.12 µM in RYGB_rat), PC ae C34:1 (10.5 µM in LS vs. 12.7 µM in OP; 2.97 µM in BWM_rat vs. 4.73 µM in RYGB_rat), PC ae C38:1 (0.17 µM in LS vs. 0.31 µM in OP; 0.19 µM in BWM_rat vs. 0.49 µM in RYGB_rat), PC ae C38:6 (5.89 µM in LS vs. 6.51 µM in OP; 1.13 µM in BWM_rat vs. 1.57 µM in RYGB_rat), PC ae C40:5 (2.32 µM in LS vs. 2.93 µM in OP; 0.59 µM in BWM_rat vs. 1.01 µM in RYGB_rat), PC ae C40:6 (2.79 µM in LS vs. 3.44 µM in OP; 1.05 µM in BWM_rat vs. 1.34 µM in RYGB_rat), PC ae C42:1 (0.25 µM in LS vs. 0.33 µM in OP; 0.25 µM in BWM_rat vs. 0.37 µM in RYGB_rat), PC ae C42:2 (0.35 µM in LS vs. 0.45 µM in OP; 0.23 µM in BWM_rat vs. 0.52 µM in RYGB_rat), PC ae C42:3 (0.44 µM in LS vs. 0.51 µM in OP; 0.43 µM in BWM_rat vs. 0.63 µM in RYGB_rat), PC ae C44:3 (0.1 µM in LS vs. 0.11 µM in OP; 0.06 µM in BWM_rat vs. 0.10 µM in RYGB_rat) and PC ae C44:6 (0.68 µM in LS vs. 0.81 µM in OP; 0.06 µM in BWM_rat vs. 0.11 µM in RYGB_rat).

Finally, two out of five sphingolipids with a significant difference in the *human* cohort were also found to be significantly different in the rodent model: SM (OH) C16:1 (2.27 µM in LS vs. 2.54 µM in OP; 0.73 µM in BWM_rat vs. 0.42 µM in RYGB_rat), and SM C16:0 (77.5 µM in LS vs. 93.4 µM in OP; 46.6 µM in BWM_rat vs. 32.2 µM in RYGB_rat). No lysophosphatidylcholines were found to overlap in both comparisons.

## 3. Discussion

The aim of our study was to identify and characterize a comprehensive metabolomic profile, which highlights the effect of bariatric surgery on the metabolome beyond weight loss. Whereas patients of the WAS intensified lifestyle group failed to lose weight significantly, patients with bariatric surgery lost a significant amount of their body weight as expected. To clarify the role of RYGB-induced body weight loss itself, the same metabolomic parameters were analyzed in rats treated with RYGB or a similar effective food restriction regime.

Examining the *human* data, twelve principal components with 57 significantly different metabolites between the LS and OP group were identified at 1-year follow-up. A characteristic metabolomic profile could be identified including two main components of differentiation. Upon closer examination of these components, sphingolipids and phosphatidylcholines were identified as the main metabolomic subgroups separating the two *human* cohorts. Both lipid groups are essential in the formation of the cell membrane and intracellular signal transduction.

Sphingolipids maintain lipid microenvironments of plasma membranes and form lipid rafts [19]. As well as their role as components of the cell membrane, sphingolipids build the carcass of myelin sheaths [25]. Recently, higher concentrations of sphingolipids together with low concentrations of BCAAs have been reported to correlate with a healthy liver phenotype [26]. The importance of sphingolipids in IR has been highlighted previously [27]. Phosphatidylcholines on the other hand build the major part of the membrane matrix [28]. Conclusively, a sensitive balance in these lipids is liable for polarization and signal transduction in the cell environment [29]. Recently, alterations in phosphatidylcholines and sphingolipids were identified as relevant, although differently regulated parts of the metabolomic profile in morbid obese as well as in patients with gastric adenocarcinoma and as key effectors of apoptosis and tumor cell growth, possibly explaining the well-known increased lifetime risk for cancer in obese subjects [30,31].

A further metabolomic subgroup included BCAA, and aromatic amino acids. The BCAAs could be identified as a valid separator of the LS and OP group at one year after randomization. As described before, BCAAs correlate with BMI and HOMA-IR [16,32,33]. Studies analyzing *human* adipose and muscle tissue in obese and diabetic conditions show a reduced activity of mitochondrial branched-chain aminotransferase. This is in accordance with the development of IR, especially if the concentration of BCAAs is elevated over a longer period of time [34]. Subjects with chronically elevated concentrations of BCAAs also have higher HOMA-IR values. Elevated leucine and valine concentrations have been associated with IR [35]. Among others, BCAAs affect the intracellular insulin signaling in *human* cells by binding to Rag GTPases, leading to the activation of mammalian target of rapamycin complex 1 (mTORC1), the activation of ribosomal S6 kinase 1 (S6K), the inhibition of insulin receptor substrate 1 (IRS-1) and the stimulation of mitochondrial dysfunction [36]. See Supplementary Figure S2 for further explanation.

Analyzing overlapping phosphatidylcholines from the metabolomic profiles as a translational approach with indirect comparison, PC aa C42:Ys have been identified as being significantly different metabolites in both comparisons, OP vs. LS and RYGB_rat vs. BWM_rat. PC aa C42:Ys, better known as lecithins, have been repeatedly mentioned in studies investigating metabolomics in obesity [16,24,28,37]. With respect to the method of analysis, the length of the two fatty acids on these phosphatidylcholines could not be delineated. However, the most frequently identified PCs are arachidic acid, lignoceric acid and arachidonic acid. All three of these fatty acids have been reported to be decreased in obese patients [12,38,39]. In the present cohort, a significant enrichment of PC aa C42:Ys was found in both surgical groups in the *human* and rat cohort. Interestingly, no significant increase in lecithins was detected in both conservatively treated groups. Therefore, an effect of RYGB on lecithins independently of weight loss can be assumed. As the weight-loss-independent effect of RYGB is a recent focus of clinical research to find effective and sustainable alternatives for the therapy of morbidly obese patients, the presented changes in the metabolome after bariatric surgery, consensually, is in accordance with previous findings [10,40].

The analysis of the metabolomics of rats with similar body weights after RYGB and diet restriction and similar food composition overcomes the limitation that weight loss effects cannot be differentiated from the effects of the bariatric intervention (i.e., changed absorbance) itself. Interestingly, lipids play a crucial role in differentiating RYGB from BWM rats, while amino acids do not facilitate the separation of these intervention groups. Therefore, changes in BCAAs after RYGB do not seem to be caused by bariatric surgery itself. A significant increase in sphingolipids as well as PC aa C42:Ys in the OP group of the WAS trial and higher levels in the BWM vs. RYGB group of our animal experiment were detected. The role of sphingolipids in obesity and IR is not fully understood and has to be investigated in further *human* studies with an adequate diet restriction paradigm [41].

Our study has several limitations. The very low sample size compared to the large number of analyzed metabolites may have hampered the interpretation of data. It was also not possible to consider the whole spectrum of confounders (e.g., smoking status) potentially influencing the metabolic spectrum. Although there was no difference in the nutrition counseling, we cannot exclude that the actual nutrition might have differed from the references within the nutrition counseling. Additionally, gender distribution differed significantly between the LS and OP group of the WAS cohort. Furthermore, a direct comparison of *human* metabolomic data with the metabolome of rats is not uncritical and therefore allows only limited conclusions regarding the underlying pathophysiological mechanism [42,43]. Nevertheless, the strength of our study was the inclusion of excellent annotated material, both from *humans* and animals that have undergone a randomly assigned intervention.

## 4. Materials and Methods

### 4.1. Patients

**Figure 7:**
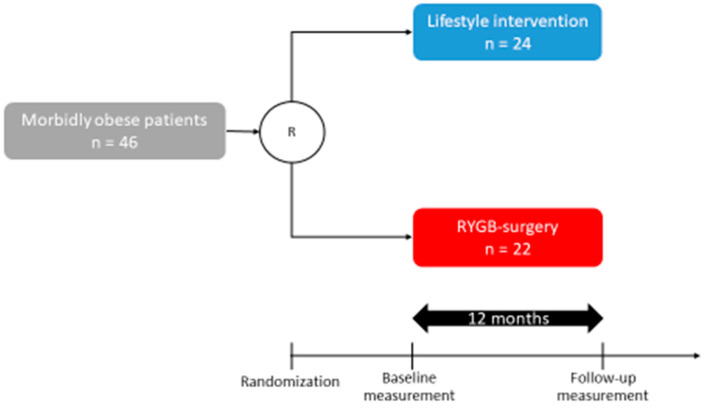
Time schedule of the WAS trial. R, randomization.

The Würzburg Adipositas Study is a randomized trial comparing the effects of RYGB vs. psychotherapy-enhanced lifestyle intervention not including calorie-limited nutrition in morbidly obese patients. Details of the design of the study are published elsewhere [22]. Twenty-four participants were randomized into intensive lifestyle modification, 22 were randomized into an RYGB-surgery group, which was performed at the department of general, visceral, transplant, vascular and pediatric surgery in the University Hospital of Wuerzburg. At twelve months after randomization, blood samples were taken for further analyses (see Figure 7). The WAS study protocol was approved by the Ethics Committee of the University Hospital of Wuerzburg (182/08). All patients provided written informed consent.

### 4.2. Animals

As described in detail elsewhere, adult male Wistar *rats* (Charles River Laboratories, n = 13) with initial body weight 323.1 ± 4.7 g, 9–10 weeks old, were group-housed in a certified facility with an ambient room temperature of 22 °C and a 12-h light/dark cycle. These animals were part of a series that was recently published [44]. Animals had free access to a high-fat diet (C1090-60 HF diet, 5228 kcal/kg; 60% calories from fat, 16% from protein and 24% from carbohydrate; Altromin, Lage, Germany) for about 6 weeks to induce obesity. The animals were then randomized into the following treatment groups and, after the respective intervention, kept on a choice of the high- and a low-fat diet (C1090-10 LF, 3514 kcal/kg; 10% calories from fat, 24% from protein and 66% from carbohydrate; Altromin). RYGB was performed on six animals. Seven animals were body-weight-matched controls, which underwent sham surgery and were then kept on chronic food restriction to induce a similar weight course as in RYGB animals. This was achieved by restricting the amount of high- and low-fat diet they consumed compared to that of RYGB_rat. As published before, animals were isoflurane-anesthetized and under butorphanol (0.1 mg/kg) analgesia for RYGB and sham operation [45]. For RYGB, a small gastric pouch 5% of the original stomach size was created, and the biliopancreatic and common limbs were made to measure 15 cm and 25 cm in length, respectively [45,46]. Four weeks after intervention, blood samples were taken in a fasted state (12 h). The local regulatory authority (Regierung von Unterfranken: 55.2-2532-2-467) approved all animal procedures.

### 4.3. Laboratory Measurements

In brief, serum samples from patients were obtained upon enrolment under standardized conditions. After an overnight fast, the blood was drawn from the cubital vein with a Safety-Multifly^®^ 21G (Sarstedt AG & Co.KG, Nümbrecht). Samples for analysis of fasting glucose were stored in S-Monovette^®^ Fluoride/EDTA (Sarstedt AG & Co.KG). Samples for the analysis of insulin and metabolome were stored in S-Monovette^®^ Serum (Sarstedt AG & Co.KG). All samples were centrifuged immediately after blood drawn at 500× *g* for 5 min. Afterwards, serum was stored at −80 °C until further analysis. HOMA-IR was calculated as follows:

HOMA-IR = insulin (fasting) (mU/l) ∗ glucose (fasting) (mg/dl)/405

Plasma samples from *rats* were collected after a 12-h fasting period under deep anesthesia shortly prior to euthanasia. Immediately after collection in tubes pretreated with a DPP-IV inhibitor (Merck), plasma was separated from the blood samples by centrifugation at 5 krpm for 10 min at 4 °C and stored at −80 °C.

### 4.4. Targeted Metabolomics

Reagents, standards and controls were prepared according to manufacturer’s instructions (Biocrates UM-P180-SCIEX-13) for mass spectrometry. Analytical columns (Acquity UPLC BEH C18 1.7 µm 2.1 mm × 75 mm, Waters) and pre-column (Acquity BEH C18 1.7 VANGUARD) from Waters (Eschborn, Germany) were used. Targeted metabolomics was performed using the AbsoluteIDQ p180 Kit (BIOCRATES Life Sciences AG, Innsbruck, Austria) [47], which complies with the EMA ‘Guideline on bioanalytical method validation’ (21 July 2011) and implies proof of reproducibility within a given error range [48]. The Biocrates AbsoluteIDQ p180 kit simultaneously identifies and quantifies 188 metabolites including 21 amino acids, 21 biogenic amines, 40 acylcarnitines, 15 sphingolipids, 14 lysophosphatidylcholines, 76 phosphatidylcholines and 1 hexose (>90% glucose). The assay-specific procedures have been described in detail elsewhere [49,50].

Serum from each sample was processed according to the manufacturer’s recommendations. In brief, 10 μL of the internal standard solution was added to each well on a filter spot of the 96-well extraction plate. After drying under a gentle stream of nitrogen, 10 μL of each serum sample, quality control (QC) sample, blank, zero sample or calibration standard, was added to the appropriate wells. The plate was then dried under a gentle stream of nitrogen. Derivatization was performed using 5% phenyl isothiocyanate (33% ethanol, 33% water, 33% pyridine). Samples were incubated for 20 min followed by extraction with methanol. Electrospray ionization (ESI)—LC-MS/MS and flow injection (FIA) MS/MS was performed on Sciex 4500QTRAP MD (SCIEX, Framingham, MA, USA). MS-system was coupled to an Agilent 1290 UHPLC-system (G4226A autosampler, infinity BinPump, G1316C column-oven, G1330B thermostat (Agilent, Santa Clara, CA, USA)).

Data were processed with Analyst Software version 1.6.2 MD (SCIEX, Framingham, MA, USA). LC- and MS/MS settings were set according to the manufacturer’s manual. MetIDQ software (5.5.4-DB 100 Boron-2623 (Biocrates, Innsbruck, Austria)) was used for validation and processing of MS data. Amino acids and biogenic amines were separated by UHPLC before injection into the mass spectrometer, while flow injection analysis was used for PCs, hexose, Cs and SMs. In this dataset, metabolites with more than 60% measurements at the limit of detection (LOD) or below lowest limit of quantification (LLOQ), and those samples with missing values for more than 60% of the metabolites were excluded from the analyses as described previously [51]. To ensure comparability of data between batch measurements, each metabolite value was normalized to four *human* reference samples included into each batch as previously described. Normal ranges for metabolites based on data described elsewhere [25,26]. In the nomenclature of presented lipids ´Cx:Y` describes the composition of the lipid chain with ´x` indicating the number of carbon atoms and ´y` the number of double bonds.

### 4.5. Statistical Analysis

Analyses were conducted using SPSS software (PASW version 25.0, SPSS Inc. Chicago, IL, USA), GraphPad Prism version 9.1.2 for Windows (GraphPad Software, La Jolla, CA, USA) and MetaboAnalyst (version 5.0, www.metaboanalyst.ca, accessed on 10 October 2022). The dataset was tested for normal distribution using the Shapiro–Wilk test. All normally distributed values are presented as mean ± SD whereas non-normally distributed data are presented as medians ± interquartile range (IQR). Outliers were defined with values >1.5 interquartile ranges (IQR) below the first quartile or above the third quartile. No outliers were identified in the *human* cohort. One *rat* was identified as an outlier and excluded from further analysis. Post hoc comparisons (Tukey–Kramer Honest Significant Difference method) were performed to control for family-wise error rates (FWER). Hierarchical clustering analysis was conducted on the final dataset to assess their putative abundances (clustering by Euclidean distance measure and Ward linkage). To visualize class differences from a multivariate dataset and to determine whether there is any cluster distinction between different groups, principal component analysis was performed. The validity of the PCA was calculated using Bartlett’s test and the Kaiser–Meyer–Olkin measure of sampling adequacy. According to the Kaiser criteria, only factors with an eigenvalue ≥ 1 were considered. The calculated principal components were defined as factor analysis components.

Clinical data of the WAS cohort was not normally distributed (Shapiro–Wilk test, *p* < 0.05). Hence, we performed Mann–Whitney tests for clinical parameters to investigate differences between groups at one-year follow-up. Data from the metabolomics followed normal distribution (Shapiro–Wilk test, *p* > 0.05). To adjust for differences at baseline, a repeated-measures analysis of covariance (ANCOVA) was performed (correcting for age and sex). 

Data in the rodent model were normally distributed (Shapiro–Wilk test, *p* > 0.05). A one-way ANOVA was performed to assess the effects of obesity treatment on body weight and the metabolome four weeks after intervention. *p*-values for the respective comparisons after correction for multiple comparisons are reported.

## 5. Conclusions

We analyzed the effects of surgical vs. lifestyle intervention on the serum metabolome of obese and insulin resistant *human* patients of the randomized controlled WAS and compared them with the results from a rodent model. As the conservatively treated *human* group failed to achieve significant weight loss, the difference in the metabolomic profile could either be associated with RYGB or weight loss. However, several metabolites were found to be significantly different in *rats* following RYGB vs. *rats* treated with an evenly effective food restriction paradigm. By indirectly comparing the metabolomic profiles of both species as a translational approach, a high number of overlapping parameters could be found. A subgroup of sphingolipids, BCAAs and phosphatidylcholines was found to be altered in both surgical groups, but not in the conservatively treated *human* and *rat* groups. Thus, these metabolites might indeed be a specific consequence of the RYGB. The significant increase in the hereby-identified lipids as well as a significant decrease in BCAA has been shown before to improve the glucose metabolism. Thereby, our investigation is in accordance with several methodically comparable studies, clarifying the complex role of RYGB, and, hence, supports the worldwide effort to investigate the weight-loss-independent effects of bariatric surgery.

## Figures and Tables

**Table 1:** Medians ± interquartile range (IQR) of baseline characteristics with standard deviation of patients enrolled in the WAS trial.

	Study Cohort (*n* = 46)	LS (*n* = 24)	OP (*n* = 22)	*p*-Value
Women	39	18	21	0.32
Men	7	6	1	<0.01
Age (years)	41.2 ± 1.5	39.3 ± 2	43.1 ± 2	0.13
Body height (cm)	172 ± 1	172 ± 2	167 ± 2	0.05
Body weight (kg)	134.9 ± 4.2	138.9 ± 3.1	128.6 ± 2.4	0.06
BMI (kg/m^2^)	45.0 ± 1.4	47.4 ± 1.2	42 ± 1	0.09
HbA1c (%)	5.9 ± 0.2	6.1 ± 0.3	5.7 ± 0.1	0.17
HOMA-IR	6.8 ± 1.3	7.1 ± 2.3	6.5 ± 0.2	0.07

**Table 2:** One-year follow-up means of 57 significantly different metabolites between LS and OP with standard deviation. *p*-values are adjusted for sex and age and corrected for multiple comparisons. C, acylcarnitine; lysoPC, lysophosphatidylcholine; PC aa, phosphatidylcholine with ester bonding; PC ae phosphatidylcholine with ether bonding; SM, sphingomyelin.

Metabolites	LS (µM)	OP (µM)	Difference (µM)	Difference (%)	*p*-Value
C3	0.43 ± 0.019	0.35 ± 0.01	0.08	22.9	<0.01
Ala	485.67 ± 21.8	392.64 ± 14.74	93.03	23.7	<0.01
Cit	25.57 ± 1.63	35.84 ± 1.55	10.27	28.7	<0.01
Gln	702 ± 30.73	834 ± 22.24	132	15.8	<0.01
Glu	73.68 ± 6.25	38.51 ± 3.50	35.17	91.3	<0.01
Gly	249.33 ± 13.55	394.68 ± 17.90	145.35	36.8	<0.01
His	82.86 ± 2.53	88.2 ± 1.37	5.34	6.1	0.04
Ile	89.55 ± 3.48	65.14 ± 2.05	24.41	37.5	<0.01
Leu	168.08 ± 4.52	121.61 ± 4.36	46.47	38.2	<0.01
Lys	296.96 ± 8.84	260.5 ± 7.09	36.46	14	<0.01
Phe	73.2 ± 1.38	56.72 ± 1.36	16.48	29.1	<0.01
Trp	65.55 ± 1.80	54.53 ± 1.76	11.02	20.2	<0.01
Tyr	82.83 ± 4.33	60.14 ± 1.68	22.69	37.7	<0.01
Val	305.04 ± 9.83	212.09 ± 7.48	92.95	43.8	<0.01
Sarcosine	1.14 ± 0.09	0.85 ± 0.06	0.29	34.1	0.04
lysoPC a C16:0	51.14 ± 2.61	58.45 ± 2.42	7.31	12.5	0.04
lysoPC a C17:0	0.74 ± 0.04	1.03 ± 0.07	0.29	28.2	<0.01
lysoPC a C18:0	12.43 ± 0.63	15.11 ± 0.89	2.68	17.7	0.03
lysoPC a C18:1	9.48 ± 0.41	15.22 ± 0.93	5.74	37.7	<0.01
lysoPC a C18:2	12.13 ± 0.70	15.17 ± 1.00	3.04	20	0.04
PC aa C32:0	16.01 ± 0.75	18.59 ± 0.65	2.58	13.9	0.03
PC aa C34:1	257 ± 16.70	283.64 ± 9.72	26.64	9.4	0.03
PC aa C36:0	1.44 ± 0.08	1.73 ± 0.1	0.29	16.8	0.04
PC aa C38:1	0.6 ± 0.08	0.97 ± 0.09	0.37	38.1	0.03
PC aa C38:5	44.18 ± 2.54	52.19 ± 2.28	8.01	15.3	0.02
PC aa C40:2	0.16 ± 0.01	0.19 ± 0.01	0.03	15.8	0.04
PC aa C40:5	6.56 ± 0.40	8.03 ± 0.45	1.47	18.3	0.03
PC aa C42:0	0.29 ± 0.01	0.35 ± 0.01	0.06	17.1	0.03
PC aa C42:1	0.13 ± 0.01	0.16 ± 0.01	0.03	18.8	<0.01
PC aa C42:2	0.13 ± 0.01	0.15 ± 0.01	0.02	13.3	0.04
PC aa C42:4	0.09 ± 0.01	0.12 ± 0.01	0.03	25	<0.01
PC aa C42:5	0.21 ± 0.01	0.28 ± 0.01	0.07	25	<0.01
PC aa C42:6	0.24 ± 0.01	0.29 ± 0.02	0.05	17.2	0.04
PC ae C32:1	3.18 ± 0.12	3.85 ± 0.13	0.67	17.4	<0.01
PC ae C32:2	0.85 ± 0.03	1.04 ± 0.04	0.19	18.3	<0.01
PC ae C34:1	10.58 ± 0.50	12.69 ± 0.44	2.11	16.6	<0.01
PC ae C34:3	6.8 ± 0.32	9.45 ± 0.5	2.65	28	<0.01
PC ae C36:5	11.52 ± 0.60	13.83 ± 0.61	2.31	16.7	0.02
PC ae C38:1	0.17 ± 0.03	0.31 ± 0.03	0.14	45.2	<0.01
PC ae C38:5	15.55 ± 0.70	17.64 ± 0.63	2.09	11.8	0.04
PC ae C38:6	5.89 ± 0.29	6.5 ± 0.26	0.61	9.4	0.04
PC ae C40:1	0.82 ± 0.04	0.98 ± 0.05	0.16	16.3	0.03
PC ae C40:5	2.32 ± 0.08	2.93 ± 0.11	0.61	20.8	<0.01
PC ae C40:6	2.79 ± 0.1	3.44 ± 0.14	0.65	18.9	<0.01
PC ae C42:1	0.26 ± 0.01	0.33 ± 0.01	0.07	21.2	<0.01
PC ae C42:2	0.35 ± 0.01	0.45 ± 0.02	0.1	22.2	<0.01
PC ae C42:3	0.44 ± 0.02	0.52 ± 0.02	0.08	15.4	0.02
PC ae C42:5	1.46 ± 0.06	1.8 ± 0.07	0.34	18.9	<0.01
PC ae C44:3	0.1 ± 0.002	0.11 ± 0.003	0.01	9.1	<0.01
PC ae C44:5	1.25 ± 0.05	1.52 ± 0.07	0.27	17.8	0.02
PC ae C44:6	0.68 ± 0.03	0.81 ± 0.03	0.13	16	0.01
SM (OH) C16:1	2.27 ± 0.09	2.54 ± 0.11	0.27	10.6	0.02
SM (OH) C22:1	5.68 ± 0.30	4.45 ± 0.24	1.23	27.6	<0.01
SM C16:0	77.54 ± 2.22	93.49 ± 2.63	15.95	17.1	<0.01
SM C24:1	21.58 ± 0.83	25.51 ± 1.01	3.93	15.4	0.02
SM C26:1	0.18 ± 0.01	0.24 ± 0.02	0.06	25	0.01
H1	5286.63 ± 337.92	4399.09 ± 101.08	887.54	20.2	0.02

**Table 3:** Means of 62 significantly different metabolites at four weeks after start of the interventions between BWM_rat and RYGB_rat with standard deviation. *p*-values are corrected for multiple comparisons. C, acylcarnitine; lysoPC, lysophosphatidylcholine; PC aa, phosphatidylcholine with ester bonding; PC ae phosphatidylcholine with ether bonding; SM, sphingomyelin.

Metabolites	BWM_rat (µM)	RYGB_rat (µM)	Difference (µM)	Difference (%)	*p*-Value
C0	49 ± 2.33	34.15 ± 2.25	14.85	43.5	<0.01
C18:1	0.099 ± 0.005	0.1465 ± 0.01	0.0475	32.4	0.02
Ala	829 ± 44.15	1044.5 ± 129.02	215.5	20.6	<0.01
Cit	73.3 ± 5.95	123.5 ± 7.36	50.2	40.6	<0.01
Gln	946 ± 35.40	735.5 ± 45.28	210.5	28.6	0.03
Glu	67 ± 6.17	99.25 ± 10.21	32.25	32.5	0.04
Lys	602 ± 26.79	722.5 ± 30.22	120.5	16.7	0.04
Trp	113 ± 7.39	82.35 ± 11.24	30.65	37.2	0.04
ADMA	0.37 ± 0.03	0.66 ± 0.03	0.29	43.9	0.04
SDMA	0.22 ± 0.01	0.32 ± 0.02	0.1	31.3	0.03
lysoPC a C16:1	3.08 ± 0.3	5.9 ± 0.83	2.82	47.8	0.03
lysoPC a C24:0	0.422 ± 0.06	0.6115 ± 0.05	0.1895	31.0	0.04
lysoPC a C26:0	0.064 ± 0.02	0.1265 ± 0.02	0.0625	49.4	0.04
PC aa C30:0	0.992 ± 0.04	1.65 ± 0.31	0.658	39.9	<0.01
PC aa C32:0	5.73 ± 0.21	11.45 ± 1.29	5.72	50.0	0.04
PC aa C32:1	3.28 ± 0.34	7.72 ± 0.47	4.44	57.5	<0.01
PC aa C32:2	0.855 ± 0.09	1.515 ± 0.2	0.66	43.6	<0.01
PC aa C32:3	0.079 ± 0.01	0.1165 ± 0.01	0.0375	32.2	0.02
PC aa C34:1	63.6 ± 4.05	109 ± 9.61	45.4	41.7	<0.01
PC aa C34:3	2.57 ± 0.28	4.57 ± 0.33	2	43.8	<0.01
PC aa C34:4	0.872 ± 0.09	1.32 ± 0.06	0.448	33.9	0.03
PC aa C36:5	3 ± 0.28	4.525 ± 0.41	1.525	33.7	0.02
PC aa C36:6	0.23 ± 0.02	0.4595 ± 0.03	0.2295	49.9	<0.01
PC aa C38:0	0.837 ± 0.03	1.74 ± 0.08	0.903	51.9	<0.01
PC aa C38:1	0.436 ± 0.12	1.084 ± 0.14	0.648	59.8	0.02
PC aa C38:5	24.3 ± 2.53	40.55 ± 4.05	16.25	40.1	0.04
PC aa C38:6	29.2 ± 3.22	44.85 ± 4.13	15.65	34.9	0.03
PC aa C40:2	0.269 ± 0.02	0.519 ± 0.05	0.25	48.2	0.02
PC aa C40:3	0.319 ± 0.01	0.6175 ± 0.05	0.2985	48.3	<0.01
PC aa C40:5	5.22 ± 0.5	7.105 ± 2.22	1.885	26.5	0.04
PC aa C42:0	0.061 ± 0.01	0.1055 ± 0.02	0.0445	42.2	0.02
PC aa C42:1	0.08 ± 0.01	0.109 ± 0.02	0.029	26.6	0.04
PC aa C42:4	0.132 ± 0.01	0.2005 ± 0.02	0.0685	34.2	<0.01
PC aa C42:5	0.132 ± 0.01	0.2805 ± 0.03	0.1485	52.9	0.03
PC aa C42:6	0.311 ± 0.03	0.4905 ± 0.08	0.1795	36.6	<0.01
PC ae C32:1	0.545 ± 0.02	0.7225 ± 0.04	0.1775	24.6	0.01
PC ae C32:2	0.096 ± 0.01	0.124 ± 0.01	0.028	22.6	0.03
PC ae C34:0	0.597 ± 0.02	0.944 ± 0.09	0.347	36.8	0.02
PC ae C34:1	2.97 ± 0.14	4.735 ± 0.26	1.765	37.3	0.03
PC ae C36:0	0.254 ± 0.01	0.372 ± 0.02	0.118	31.7	0.02
PC ae C36:1	1.21 ± 0.09	2.08 ± 0.18	0.87	41.8	0.02
PC ae C36:3	0.545 ± 0.06	0.707 ± 0.05	0.162	22.9	0.04
PC ae C38:0	0.566 ± 0.10	0.8355 ± 0.16	0.2695	32.3	<0.01
PC ae C38:1	0.193 ± 0.02	0.4965 ± 0.06	0.3035	61.1	0.02
PC ae C38:3	0.506 ± 0.04	0.6865 ± 0.04	0.1805	26.3	0.04
PC ae C38:6	1.13 ± 0.09	1.575 ± 0.12	0.445	28.3	0.04
PC ae C40:3	0.225 ± 0.01	0.2915 ± 0.03	0.0665	22.8	0.04
PC ae C40:5	0.593 ± 0.06	1.014 ± 0.09	0.421	41.5	0.02
PC ae C40:6	1.05 ± 0.09	1.345 ± 0.15	0.295	21.9	0.04
PC ae C42:1	0.262 ± 0.02	0.373 ± 0.04	0.111	29.8	0.04
PC ae C42:2	0.238 ± 0.03	0.521 ± 0.08	0.283	54.3	0.03
PC ae C42:3	0.368 ± 0.05	0.6365 ± 0.09	0.2685	42.2	0.04
PC ae C44:3	0.067 ± 0.004	0.102 ± 0.01	0.035	34.3	0.02
PC ae C44:6	0.059 ± 0.01	0.1155 ± 0.01	0.0565	48.9	<0.01
SM (OH) C14:1	1.25 ± 0.04	0.7465 ± 0.07	0.5035	67.4	<0.01
SM (OH) C16:1	0.771 ± 0.05	0.3935 ± 0.03	0.3775	95.9	<0.01
SM (OH) C24:1	1.94 ± 0.1	0.7155 ± 0.15	1.2245	171.1	<0.01
SM C16:0	49 ± 2.54	33.95 ± 2.27	15.05	44.3	<0.01
SM C16:1	3.89 ± 0.18	2.555 ± 0.3	1.335	52.3	<0.01
SM C18:1	2.84 ± 0.23	1.37 ± 0.18	1.47	107.3	0.02
SM C20:2	0.095 ± 0.01	0.0455 ± 0.02	0.0495	108.8	0.04
SM C26:0	0.148 ± 0.02	0.074 ± 0.01	0.074	100.0	<0.01

**Table 4:** Eighty-one significantly different metabolites subsequently in LS vs. OP of the WAS cohort at one-year follow-up and in animals treated with RYGB vs. diet-restricted animals of the rat cohort four weeks after start of the interventions. Thirty-one overlapping metabolites found to be significant in both comparisons are printed in bold. Arrows indicate if the metabolite is increased or decreased in the respective surgical group in comparison the conservative group. C, acylcarnitine; lysoPC, lysophosphatidylcholine; PC aa, phosphatidylcholine with ester bonding; PC ae phosphatidylcholine with ether bonding; SM, sphingolipid.

Amino-Acids		Lysophosphatidylcholines		Phosphatidylcholines—Ester		Phosphatidylcholines—Ether		Sphingolipids	
WAS	Rat	WAS	Rat	WAS	Rat	WAS	Rat	WAS	Rat
Gly ↑		lysoPC a C16:0 ↑		PC aa C36:0 ↑		PC ae C34:3 ↑		SM (OH) C22:1 ↓	
His ↑		lysoPC a C17:0 ↑		PC aa C42:2 ↑		PC ae C36:5 ↑		SM C24:1 ↑	
Ile ↓		lysoPC a C18:0 ↑		**PC aa C32:0 ↑**	**↑ PC aa C32:0**	PC ae C38:5 ↑		SM C26:1 ↑	
Leu ↓		lysoPC a C18:1 ↑		**PC aa C34:1 ↑**	**↑ PC aa C34:1**	PC ae C40:1 ↑		**SM (OH) C16:1 ↑**	**↓ SM (OH) C16:1**
Val ↓		lysoPC a C18:2 ↑		**PC aa C38:1 ↑**	**↑ PC aa C38:1**	PC ae C42:5 ↑		**SM C16:0 ↑**	**↓ SM C16:0**
Phe ↓			↑ lysoPC a C16:1	**PC aa C38:5 ↑**	**↑ PC aa C38:5**	PC ae C44:5 ↑			↓ SM (OH) C14:1
Tyr ↓			↑ lysoPC a C24:0	**PC aa C40:2 ↑**	**↑ PC aa C40:2**	**PC ae C32:1 ↑**	**↑ PC ae C32:1**		↓ SM (OH) C24:1
**Trp ↓**	**↓ Trp**		↑ lysoPC a C26:0	**PC aa C40:5 ↑**	**↑ PC aa C40:5**	**PC ae C32:2 ↑**	**↑ PC ae C32:2**		↓ SM C16:1
**Lys ↓**	**↑ Lys**			**PC aa C42:0 ↑**	**↑ PC aa C42:0**	**PC ae C34:1 ↑**	**↑ PC ae C34:1**		↓ SM C18:1
**Ala ↓**	**↑ Ala**			**PC aa C42:1 ↑**	**↑ PC aa C42:1**	**PC ae C38:1 ↑**	**↑ PC ae C38:1**		↓ SM C20:2
**Cit ↑**	**↑ Cit**			**PC aa C42:4 ↑**	**↑ PC aa C42:4**	**PC ae C38:6 ↑**	**↑ PC ae C38:6**		↓ SM C26:0
**Gln ↑**	**↓ Gln**			**PC aa C42:5 ↑**	**↑ PC aa C42:5**	**PC ae C40:5 ↑**	**↑ PC ae C40:5**		
**Glu ↓**	**↑ Glu**			**PC aa C42:6 ↑**	**↑ PC aa C42:6**	**PC ae C40:6 ↑**	**↑ PC ae C40:6**		
					↑ PC aa C30:0	**PC ae C42:1 ↑**	**↑ PC ae C42:1**		
					↑ PC aa C32:1	**PC ae C42:2 ↑**	**↑ PC ae C42:2**		
					↑ PC aa C32:2	**PC ae C42:3 ↑**	**↑ PC ae C42:3**		
					↑ PC aa C32:3	**PC ae C44:3 ↑**	**↑ PC ae C44:3**		
					↑ PC aa C34:3	**PC ae C44:6 ↑**	**↑ PC ae C44:6**		
					↑ PC aa C34:4		↑ PC ae C34:0		
					↑ PC aa C36:5		↑ PC ae C36:0		
					↑ PC aa C36:6		↑ PC ae C36:1		
					↑ PC aa C38:0		↑ PC ae C36:3		
					↑ PC aa C38:6		↑ PC ae C38:0		
					↑ PC aa C40:3		↑ PC ae C38:3		
							↑ PC ae C40:3		

## Data Availability

Data are contained within the article or Supplementary Material. The raw data are available on request from the corresponding author.

## Supplementary Materials

The following supporting information can be downloaded at: https://www.mdpi.com/article/10.3390/ijms24032354/s1, Figure S1: Unsupervised principal component analysis of 13 significantly different metabolites between lifestyle intervention and surgery group in the WAS cohort at baseline visit.; Figure S2: Intracellular BCAA pathway leading to insulin resistance.
