# Supplementary material for: Changes in Plasma Metabolomic Profile Following Bariatric Surgery, Lifestyle Intervention or Diet Restriction—Insights from Human and Rat Studies
Source: Int J Mol Sci. 2023 Jan 25;24(3):2354. doi: 10.3390/ijms24032354 (PMC9916678; doi:10.3390/ijms24032354)
Supplement: Supplementary file 1 [file ijms-24-02354-s001.zip › ijms-2036278 - supplementary.pdf]

Changes in plasma metabolomic profile following bariatric surgery, lifestyle intervention or diet restriction – insights from human and rat studies

Supplementary Materials

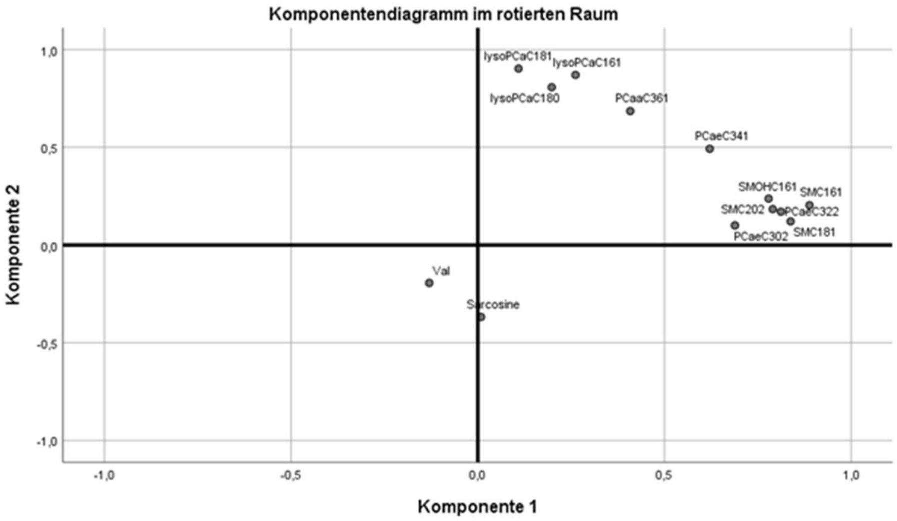

Supplementary Figure S1: Unsupervised principal component analysis of 13 significantly different metabolites between lifestyle intervention and surgery group in the WAS cohort at baseline visit. The first component explains 37 % of the variation, and the second component 23 %. C, acylcarnitine; lysoPC, lysophosphatidylcholine; PC aa, phosphatidylcholine with ester bonding; PC ae phosphatidylcholine with ether bonding; SM, sphingolipid.

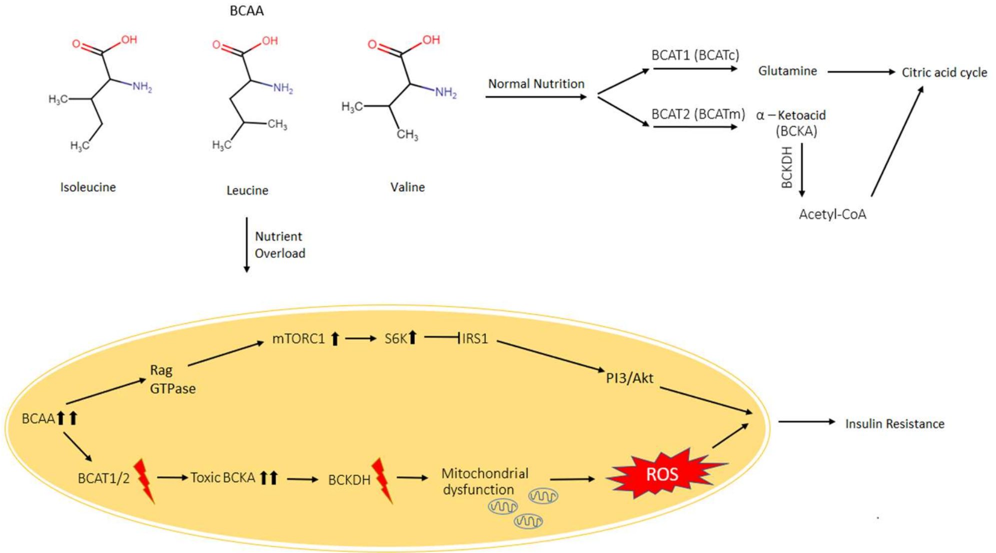

Supplementary Figure S2: Intracellular BCAA pathway leading to insulin resistance. Branched-chain amino acids are mainly metabolized in the cytosol (BCAT1) and mitochondria (BCAT2) of muscle cells. The resulting acetyl-CoA is a substrate for the mitochondrial respiratory chain. Increasing levels of BCAAs accumulate as toxic branched-chain ketoacids and result in the accumulation of oxidative species. Additionally, high levels of BCAAs act as signal molecules stimulating mTORC1 and S6K. Subsequently, insulin receptor substrate 1 is inhibited. BCAA, branched-chain amino acids; BCAT1, branched-chain amino acid transaminase 1; BCATc, branched-chain amino acid transferase cytosolic; BCAT2, branched-chain amino acid transaminase 2; BCATm, branched-chain amino acid transaminase mitochondrial; BCKA, branched-chain ketoacid; BCKDH, branched-chain ketoacid dehydrogenase; mTORC1, mammalian target of rapamycin complex 1; S6K, Serine 6 kinase; IRS1, insulin receptor subtract 1; PI3/Akt, phosphatidylinositol-3-kinase/Akt pathway; ROS, reactive oxygen species.

Leucine has been demonstrated to have greater effect on the intracellular insulin signalling pathways than valine and isoleucine [11]. Acylcarnitines mostly derive from branched-chain amino acid metabolism and form obligate cofactors of mitochondrial fatty acid β-oxidation on the outer mitochondrial membrane. On the inner mitochondrial membrane, acylcarnitines are converted to Acyl-CoA. Finally, new substrates enter the citric acid cycle to generate NADH and FADH2 to supply the electron transport chain [12]. An alteration of BCAAs and acylcarnitines in serum may reflect an imbalance in energy homeostasis as seen in obese subjects. As an effect of such an imbalance, oxidative species accumulate on the outer as well as the inner mitochondrial membrane. Higher serum levels of acylcarnitines and branched-chain amino acids may partially explain mitochondrial dysfunction and subsequently insulin resistance in higher grades of obesity. Intermediates of the catabolism of BCAAs, e.g. branched-chain ketoacids accumulate in insulin resistant subjects and inhibit the mitochondrial respiratory chain [31].
